# Supporting families and caregivers of children with disabilities through a parent peer mentor (PPM): experiences from a patient-oriented research network

**DOI:** 10.1186/s40900-023-00481-y

**Published:** 2023-09-08

**Authors:** Sakiko Yamaguchi, Carrie Costello, Corinne Lalonde, Sharon McCarry, Annette Majnemer, Keiko Shikako

**Affiliations:** 1 School of Physical and Occupational Therapy, Faculty of Medicine and Health Sciences, McGill University, Montreal, Canada; 2 CHILD-BRIGHT Patient-Oriented Research Network, Montreal, Canada; 3 Research Institute of the McGill University Health Centre, Montreal, Canada; 4 Centre for Interdisciplinary Research in Rehabilitation (CRIR) | MAB-Mackay, 3500, Blv Décarie, Montreal, QC H4A 3J5 Canada

**Keywords:** Parent-partner, Parent peer mentor, Patient-oriented research, Patient engagement

## Abstract

**Background:**

The CHILD-BRIGHT Network created a parent peer mentor (PPM) role to support other parents who were engaging as partners in the different research projects and activities of the network. We aim to describe how a PPM functioned to support parent-partners of children with disabilities in research projects within the Network.

**Methods:**

In this case study, the PPM approached 50 parent-partners and scheduled a 1-on-1 initial telephone call to offer support for any issues arising. When consent was provided, the PPM recorded interactions with network parent-partners in a communication report in an Excel form. Also, verbatim transcription from one in-depth interview with the PPM was included for data analysis using qualitative description. The Guidance for Reporting Involvement of Patients and the Public (GRIPP2-SF) was used to report on involvement of patient-partners.

**Results:**

A total of 55 interactions between 25 parent-partners and the PPM were documented between May 2018 and June 2021. The PPM’s support and liaison role contributed to adaptation of meeting schedules for parent-partners, amendment of the compensation guidelines, and ensuring that internal surveys and the newsletter were more accessible and engaging. The PPM also facilitated community-building by keeping parent-partners connected with researchers in the Network. Families and caregivers in the Network were comfortable sharing their experiences and emotions with the PPM who was also a parent herself, allowing researchers and the Network to learn more about parents’ experiences in partnering with them and how to improve engagement.

**Conclusions:**

We highlight the important complementary role that a PPM can play in enhancing patient engagement in research by better understanding the experiences and needs of parent-partners.

**Supplementary Information:**

The online version contains supplementary material available at 10.1186/s40900-023-00481-y.

## Abstract (plain language summary)

In this paper, we add new insights into the complementary role of the Parent Peer Mentor (PPM) in promoting optimal patient engagement practices in a national patient-oriented research network. The PPM approached 50 parent-partners and scheduled a 1-on-1 initial telephone call to offer support for any issues arising. We analyzed the recorded interactions between the PPM and the network’s parent-partners of children with brain-based disabilities as well as an interview with the PPM. The PPM’s liaison role contributed to establishing reciprocal connections with parent-partners in a nation-wide research network. The perceived impacts at the individual level included: (1) parents felt more connected to the PPM and were comfortable sharing their experiences and emotions, and (2) researchers learned more about parents’ experiences in partnering with them and were able to address the issues raised such as adjustment of the meeting schedule and clarification of roles within the research team. At the Network level, adjustments were made based on feedback from the PPM to include structural adaptations to the compensation guidelines and use of lay language in our communications to patient-partners. Community-building and authentic partnerships were enhanced by the increased understanding of the experiences of patient-partners.

**Supplementary Information:**

The online version contains supplementary material available at 10.1186/s40900-023-00481-y.

## Background

Families and caregivers of children with disabilities play a crucial role in providing necessary services and support to their child. However, access to information and needed services is one of the major challenges that arise while raising a child with disabilities [36, 39]. Online peer mentorship may help address this challenge by providing information and social support and opportunities to speak to those who can relate to certain experiences [27, 38].

In health care research, patient-partners, defined as people with lived experience of a health condition and/or health care system, including children with a health condition as well as parents and informal caregivers, are increasingly playing active roles as collaborators and advisors, on research teams [40]. Engagement with patient-partners is increasingly recognized as an essential aspect of patient-oriented research [15]. The Canadian Institutes of Health Research has developed a Strategy for Patient-Oriented Research (SPOR), which envisions patient involvement in health research teams “as much and as meaningfully as possible” to develop research protocols and generate knowledge that is more relevant, and is more likely to improve health outcomes and enhance the health care system [8]. Engagement of patient-partners in research teams ensures that recruitment materials and strategies are optimized, that interventions are feasible and acceptable to patients, and that outcome measures are more meaningful and relevant to patients and families. There are a range of expected impacts, including patient empowerment, rapid knowledge translation and uptake of results, and greater accountability and transparency in health research. Patient engagement also responds to the moral obligation under the premise “nothing about us, without us” to facilitate the right of involvement of patients and other interested parties [15, 16, 29]. In this milieu, initiatives to support the development of patient-partnerships, such as capacity building through development of curriculums and training materials, are increasingly undertaken [3, 18, 28].

The CHILD-BRIGHT Network is an innovative pan-Canadian network that aims to improve life outcomes for children with brain-based developmental disabilities and their families. This Network was funded by the Canadian Institutes of Health Research SPOR program, and therefore patient-partners (youth with disabilities, parents/caregivers of children with disabilities) are actively engaged as partners in all research projects and the activities of the Network as a whole. Meaningful partnerships with patient-partners are expected to assist the Network in setting research priorities and developing innovative and timely research projects aimed at promoting brain and child development, optimizing mental health and well-being of children and families, and redesigning health care delivery to address service gaps. Using family and child-focused approaches, novel interventions have been created and tested to optimize development, promote health outcomes, and deliver responsive and supportive services since 2016 with funding by the Canadian Institutes of Health Research (CIHR) under Canada’s SPOR, and 27 funding partners from public and private sectors across Canada.

In order to broaden engagement of patient-partners (i.e., youth with developmental disabilities, parents/caregivers of children with disabilities), several strategic actions have been adopted. For instance, an online matching tool (identifying projects that required patient-partner input, and patient-partners who were willing to participate in research) was created in 2017 to match interest with need, and make opportunities more widely available for potential patient-partners [9]. In addition, the Citizen Engagement Council provided consultations to researchers who were struggling with their engagement of patient-partners. Despite these efforts made to improve patient engagement in the Network, the need to provide structured peer (parent to parent) mentorship for parent-partners was identified as a strategy to enhance and maintain engagement, based on experiences and feedback from patient-partners during the first year of the Network development. This additional one-to-one support and mentorship was meant to complement patient-oriented research training of researchers and patient-partners as well as consultation to research teams on authentic engagement of patient-partners.

Considering the context where most of the parent-partners were involved in research teams for the first time and parent-partners participated from different places across the country, the Network created a parent peer mentor (PPM) role to support parent-partners in our patient-oriented research Network. The peer mentorship can be defined as “a situation where a person who has lived through a specific experience (peer mentor) and a person (or a small group) who is new to that experience (peer mentee) connect in a structured setting or context” ([6, 38], p. 133). While strong team communication contributes to team development and fosters partnership, power imbalances between patient-partners and researchers, use of jargons, and time pressures on the research process can be barriers to open communication [4, 5]. In order to address these engagement barriers, the specific objectives of PPM included: (1) providing additional open channels of communication to ascertain if there are challenges with patient engagement in research; (2) identifying the needs of parent-partners engaging in research projects; and (3) identifying strategies that facilitate engagement at the research team/Network levels.

In this paper, we aimed to describe how a PPM functioned to support parent-partners of children with disabilities and worked to understand, document, and improve their experiences as partners in research projects within the CHILD-BRIGHT Network. Furthermore, we describe how a PPM can inform more optimal engagement practices in a research network.

## Methodology

A case study design is adopted to understand the phenomenon of parent-peer mentorship in the context of patient-oriented research network where parent-partners of children with disabilities are actively involved [2, 43]. In this case study, a research team that consisted of researchers and parent-partners collected information about the experiences of the PPM using multiple data collection methods over a sustained period of time [35]. We also used the Guidance for Reporting Involvement of Patients and the Public, GRIPP2-SF, to report on the involvement of our patient-partners in this study (see Additional file 1).

### Creating a PPM role in a patient-oriented research network

The CHILD-BRIGHT Network is part of the Canadian Institutes of Health Research Patient-Oriented Research Strategy. The Network supported 13 innovative research projects within its Research Program. It also had a Knowledge Translation Program, a Training Program, and a Citizen Engagement Council (CEC)—all of which had a total of 53 parent-partners including eight parent-partners who were on various committees in 2021. The PPM position was created two years after the beginning of the Network’s launch to respond to a necessity identified by the CEC and the Network leadership to create a more individualized approach in supporting and learning from the parents involved in the Network projects and programs. The CEC took the lead in defining the role for a PPM and participated in the assessment and interviews of candidates. The PPM was required to have experience in research, and more specifically POR and skills in listening and facilitation. Their role was to connect with other participants of studies to interview them on their experiences, help them to develop more skills to be more confident with their contributions, and collect the information.

Initially, the first PPM (Melanie [pseudonym]) assigned to the role was a mother of three children with brain-based developmental disabilities. She had previous experience in engaging in research and as a family advisor at a hospital-based research institute and actively participated in activities within the childhood disability community.

Melanie was in the role for one year, shaping some of the initial conversations with families and structure to collect feedback and communicate with the network. Building on the contributions and lessons learned from Melanie, another parent, who was a parent-partner in one of the Network research projects, stepped into the position. The second PPM, Amy (pseudonym) was a mother of three children, one of which has brain-based developmental disabilities. The position was refined and formalized, and a consultant contract was established with the Network for Amy to work one day/week in this role. Amy had previous experience in facilitating support groups for families and enjoyed the coaching role. Her major responsibility, outlined in the Terms of Reference that were mutually designed by the CEC members and the researchers, was to inform, guide, and support other parents participating as partners in the Network and become familiar with their needs to fulfill their roles. She also answered questions and informed parent-partners about available resources and strategies that were perceived as successful for engagement in other research projects and Networks. As part of her role, she took part in informal conversations with individual parents and also small groups of parents involved in the Network, to provide support as necessary.

### Data collection by the PPM

The PPM approached 50 parent-partners by email who were involved in the different research projects, programs and governance of the Network, to schedule a 1-on-1 initial telephone call. The objective was to introduce herself, explain her role as a PPM, talk about their experiences as a patient-partner in research and offer her support for any issues arising using the guiding questions (Table 1). When Amy came in, she consulted with the Central Office of the Network to prioritize projects that experienced some challenges with engagement of parents in their research teams, or teams for which limited information was available regarding their experiences with patient-partners.

**Table 1:** Parent peer mentorship guiding questions

1	How are you?
2	What’s working well today/this week/this month?
3	What’s not working well; or what do you think could work better?
4	Do you have everything (resources/tools) you need to do your work? If not, what would help?
5	How can I help you in your role with project/program X?
6	Is there anything else that would be helpful for me to know?

At the initial contact, the PPM asked each parent-partner for consent to share the data she was collecting in a blind fashion, so as to conceal the source. The PPM made clear that parent-partners receive mentorship support even if the consent for data sharing was not provided. The data was aggregated with key successes and challenges identified, to inform the network’s future patient engagement strategies. Once consent was obtained, the PPM entered the raw data taken from her notes during the interaction into an Excel form (Communication Report database) created by the Network leadership in partnership with the PPM. The responses to the guiding questions were entered into Communication Report database shortly after the interaction. The variables collected in the Communication Report included: date of interaction; mode of interaction, duration; the need for follow-up to respond to an inquiry; discussion topics; resources shared; and challenges, issues, or concerns mentioned.

To maintain confidentiality, a code was used instead of names of parent-partners. For follow-up meetings, the same code was used to longitudinally link collected data. Subsequent phone calls or one-on-one interactions were arranged at a frequency agreed upon by the PPM and the parent-partner who made the request. Several parent-partners elected not to meet with the PPM; this was not a requirement but rather, meant to support those in need.

### Interview with the PPM

Based on the preliminary analysis of the data within the Communication Report, a researcher (SY) from the Knowledge Translation program of the Network conducted a one-hour semi-structured interview with Amy to gain a better understanding of her experiences as the PPM. Questions included highlights, challenges, and lessons learned from the two-year experience as a PPM (Additional file 2).

### Ethical considerations

The McGill University Health Centre Research Ethics Board provided ethics approval (2018-4475) for this study. Once the PPM anonymized any identifiable information of the parent-partners (e.g., names of individuals, name of research project) when entering the data into the database, she maintained the database and personal notes in a secure OneDrive password‐protected account.

### Data analysis

In this study, we employed qualitative description to describe the PPM’s role in patient engagement in a research Network using language from the collected data [31, 37]. Data points were the notes from interactions collected by the PPM, Communication Reports database, and the interview transcript. The qualitative data from these documents were coded to conduct content analysis using NVivo12 software [23, 37]. The ongoing analysis of results were shared and reviewed internally with co-authors, who were CEC members including the PPM, parent-partner, and program coordinator, the Network scientific director, and the KT program academic co-lead, to build consensus on interpretation of the emerging findings, add missing perspectives, and draw conclusions. In addition, other Network documents, such as annual Report to Community and stakeholder engagement survey reports, were reviewed to triangulate findings from the analysis.

## Results

### The PPM interactions with parent-partners

Twenty-five (25) parent-partners provided their verbal consent to allow the PPM to take notes of their interactions (one or more) with the PPM between May 2018 and June 2021 and entered the information into Communication Report database. Half of the parent-partners received parent peer mentorship support, which we believe is a reasonable and adequate proportion, considering that parent-partners have a busy schedule and their contribution to the research projects and to the network are already a pressure on their time. Those who did not provide their consent (n = 25) still received the mentorship support if they wished. The communication between the PPM and parent-partners is summarized in Table 2.

**Table 2:** Overview of the provided parent peer mentorship support

Number of the total parent-partners in the Network (2021)	53
Number of parent-partners that the PPM contacted (May 2018–June 2021)	50
Number of parent-partners whose communication with the PPM was recorded in the database	25
Frequency of the PPM support received by parent-partners	
Once	10
Twice	9
Three times	3
Five times	2
Nine times	1
Format of receiving the PPM support	
Phone	38
E-mail	13
In-person meetings	3
Video call	1

Among the total 55 recorded interactions, 38 were completed by phone, 13 by emails, three by in-person meeting, and one by video call, with the average interaction time (except e-mail) being 45 min. Except for the summer period (July–August), the PPM reached out to approximately eight to eleven parent-partners every two months and arranged meetings and/or calls as needed. Ten parent-partners communicated with the PPM only once, nine partners twice, three partners three times, two partners five times, and one partner nine times.

Even though the division of tasks between parent-partner support, network improvement and discussions with research teams was not measured, one day per week was sufficient to reach out to parent-partners and carry out the necessary follow-up with research teams/the Network.

### Liaison between researchers and parent-partners

One major role was to act as a liaison with the research teams to report practices, concerns, and suggestions shared by parent-partners. Amy reflected that she saw her role predominantly as a Parent Liaison, not a Mentor, as not every parent at the Network needed “mentoring”. By playing the liaison role, Amy contributed to addressing emerging challenges and issues that could hamper trust-building and meaningful engagement in particular research projects. For example, some parent-partners raised issues with meeting schedules and formats, and suggested it was easier to share their engagement experiences more openly 1:1, rather than in large group by teleconference.

“Research team [is] amazing, [but] work schedule prevents [parents] from attending the group meetings…Would like one on one calls more, that has never been offered” (Communication report)

“Researcher went through results at a meeting, but parents did not understand and there was no check-in to make sure people were understanding…Some issues of moderation during calls with parents: Some parents take over and other quieter parents pull back. So even though they would like to speak, they don’t always feel they can” (Communication report)

When issues arose, Amy asked how the parent-partner would like to proceed. Unless the parent-partner felt comfortable approaching the team on their own or did not want to issue addressed with the team, she approached the research team with permission from the parent-partner. Then, Amy reported back to the parent-partner the results of the conversation. In response to the parent-partners’ concerns, projects made efforts to adjust their meeting times and formats to better accommodate their parent-partners. Indeed, several of the principal investigators and research coordinators would reach to patient-partners for feedback either individually or in a small group, but not with the larger research team as this was less intimidating and often preferred.

When parent-partners reported their confusion about their roles in the research project and researchers’ questions and consent documents, Amy also followed up with researchers.

“…long questionnaires and complicated consent forms should not be the first thing in a relationship with families, especially when families are in traumatic situations” (Communication report)

She suggested that research teams should clarify expectations for patient-partners at the beginning and each phase of the research cycle (i.e., ethics submission, recruitment, data collection, data analysis, manuscript preparation, knowledge translation). For e-mail communications from researchers, Amy shared a suggestion from a parent-partner that e-mails should very clearly state at the top or in the subject heading if an action is required on the part of the parent-partner. Also, she provided suggestions to parent-partners in terms of how to suggest ideas to the research team and to ask to be informed about the progress of the project throughout the research cycle.

### Listening and creating a safe space

Creating a safe space for parent-partners to share not only positive experiences and suggestions but also difficult feelings was a crucial aspect of the PPM’s liaison role. Several parent-partners were initially “feeling bad reaching out to researchers [as] they feel like [they are] pestering” (Communication Report). Amy’s ability to listen rather than direct the conversation was successful in “creating the environment where it is OK to talk about bad things” by challenging “the assumption that we have to say everything is fine” (interview). Even parent-partners who were very comfortable with researchers “needed someone outside of the ‘research team’ to confide in and speak about issues with” (Communication Report). Amy also stated,

“Having someone who is a sort of go-to person or people can talk to, if the relationship with research teams is not going well, having that person identified, I think, makes people feel safer” (interview).

On occasion, a subset of parent-partners exhibited emotions such as frustration and anger, although many also shared their positive experiences of belonging, feeling part of something meaningful and contributing for a greater good.

Amy believes that parent-partners found her easier to connect with because she had similar experiences as a peer, which can generate a more “immediate trust” (interview). On occasions, the conversation extended beyond involvement in the research projects as one parent-partner shared, “living and fighting everyday for our children can be too much sometimes” (Communication report). Particularly when the COVID-19 pandemic impacted the everyday life of parent-partners and their children, parent-partners shared personal challenges related to family issues and coping during the pandemic with regard to service access and mental health. Amy provided emotional support by listening to parent-partners and shared resources if needed.

### Improving the research partnership development

**Fig. 1:**
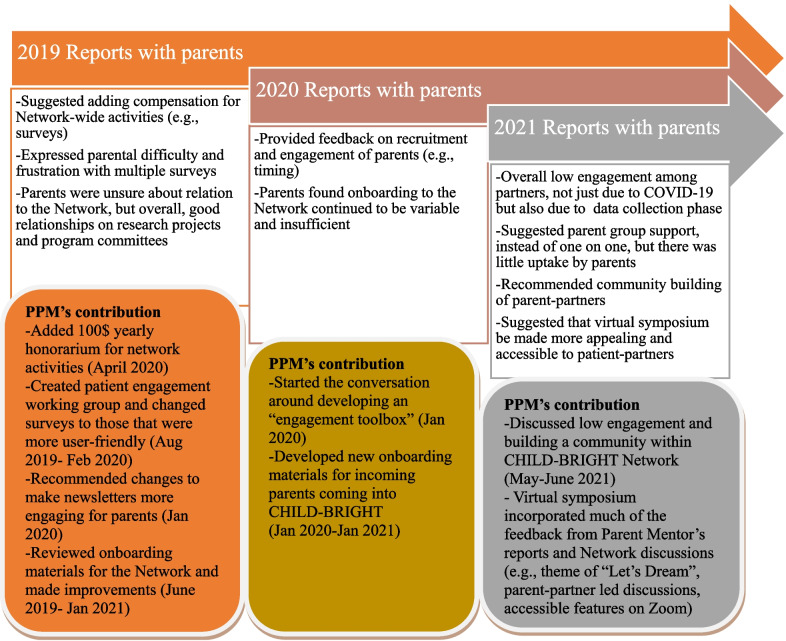
Parent Peer Mentor (PPM) at a glance

The PPM’s liaison role helped to provide feedback to the Network on aspects of patient engagement that were positive such as patient-partners’ sentiments of: “feel[ing] engaged,” “proud to be part of [the project]”, and “projects are worthwhile and exciting”. On the other hand, the PPM also identified room for improvement in the Network-level support through their conversations with parent-partners. One important contribution of the PPM role was an amendment to the Network’s patient-partner compensation guidelines (Fig. 1). Specifically, many parent-partners understood that they had “signed onto a research project which is very clear about expectation and time commitments and the researcher is very respectful of the parents’ time and expertise” (Communication Report), but that other work for the Network, such as completing surveys and conducting interviews, should be separately compensated.

“Surveys and speaking with the parent peer mentor or communications or anything like that is not outlined anywhere in the compensation guidelines” (Communication Report)

Amy brought this issue to the attention of the CEC and the Executive Committee. The Network amended its guidelines to include separate compensation for patient-partners participating in Network-level activities beyond their initial project or program or committee commitment [12] (Additional file 3).

Another contribution was to make the Network-level surveys and newsletters more accessible to parent-partners. While parent-partners were asked to review surveys for studies, the Network conducted the stakeholder engagement surveys (e.g., patient engagement measurement, sociodemographic profile, engagement experiences during the pandemic) annually beginning 2018, to evaluate patient engagement experiences from the perspectives of researchers, patient-partners (parents and youth), trainees, and committee members. Initially, Community-Based Participatory Research (CBPR) and the Community Impacts of Research Oriented Partnerships online questionnaires were administered. However, several parent-partners reported to the PPM that the language was difficult to understand in one of the measures and that one survey was less relevant to their experiences.

Amy shared the feedback with the Network’s Measuring Patient Engagement Working Group, and one of the measures was replaced by another patient engagement measure (Public and Patient Engagement Evaluation Tool) that was more user-friendly and relevant to the Network context, and the corresponding changes were implemented into the next cycle. The improvement was noted by one parent-partner who reported that “Survey was easy to complete and submit” (Communication Report).

In addition, the newsletter, which was prepared by the Communications team and sent bi-monthly to all the Network members, including parents, researchers, trainees and community members, became more accessible. After receiving some comments from parent-partners who found the newsletter “not relevant,” “not catch attention,” and “much about research” (Communication report), Amy shared the parent-partners’ suggestions on more visuals and infographics instead of texts, and more learning opportunities on advocacy with the Network Communication Manager. After ongoing efforts were made to enhance accessibility, one parent-partner stated “Newsletter [is] clear and succinct” (Communication Report).

### Supporting community-building

The PPM’s liaison role was crucial in facilitating community-building by fostering stronger connections between parent-partners and researchers in the Network. Amy found that many parent-partners were not aware of being connected to a larger network even if they were well connected to research projects. This realization led to the development of new onboarding materials for parent-partners who joined the Network.

As some parent-partners expressed that they “would like more opportunity to connect with other parents” and “would like to support other parents new to research” (Communication Report), Amy felt “people needed support; they needed to feel a sense of community” particularly at the Network level, while each research project was “a sort of little bubble” (interview). Parent-partners who were participating in Network’s 13 research studies across Canada did not necessarily have a community in the same way as researchers who already had an established informal connection to garner support from colleagues and their institutions. Indeed, most of the 50 parent-partners who were onboard at the beginning of the research projects were involved in research teams for the first time.

In early March 2021 when the pandemic still had a major impact on the daily life of parent-partners, most research projects were in the data collection phase with little involvement of the research team as a whole. A subset of parents shared their feelings of being disconnected while not being updated about the research findings and not knowing their role in the data collection phase of the research. This may also reflect the experiences and challenges of the COVID-19 pandemic and many consequences both for parents’ availability to engage in research (e.g., due to remote work) and for researchers (e.g., due to study interruptions and lack of team building in-person experiences). In this context, Amy noted lower engagement among parent-partners whose “anger, frustration [of being] overwhelmed spilt over into [questions]: “Why am I doing this?,” “Why am I here not getting anything out of this?” (interview). While noting “nobody had really good answers for how to build community when asked about that” (interview), Amy reflected “there is much for us to learn from these experiences and also take all of this in the context of a time of a pandemic and a resetting of priorities based on what is happening in the world and where we are in the research process for many projects” (interview).

Based on this reflection, Amy suggested to the CEC that each research team create a short video clip for a virtual symposium to be held in May 2021. The suggestion was adopted under the theme of “Let’s Dream Together.” Research teams created a three-minute video clip, many of which included parent-partners, and articulated the goals of the project and hopes for the future. Amy believed that co-creating the video between researchers and parent-partners was “important for all of us to remember why we are doing [research]” (interview).

## Discussion

The initial expectations of the Parent Peer Mentor, to reach out to parent-partners and inform, guide, and support them through informal conversations, expanded to a role of liaison with the Network (Table 3). Early in the PPM role, Amy realized what many parents needed was someone to liaise with the research projects and the network as a whole, to allow for recalibration and changes in ‘real time’ to the engagement process based on parents’ needs and suggestions.

**Table 3:** Overview of the PPM’s roles and benefits

Level	Roles	Benefits
Research team	Receive feedback and suggestions from parent-partners Identify parent-partners’ needs and issues Liaise between parent-partners and researchers Create a safe space	Improvement of research team environment (e.g., meeting schedule and format, clarification of roles) Immediate connection and trust building through the lived experience Provision of emotional support
Network	Liaise between parent-partners and the Network Support community-building	Informal monitoring of engagement Improvement of Network-level communication and support (e.g., accessible newsletter, compensation)

Parent-partners’ experiences of engagement in this patient-oriented research Network were wide-ranging, from highly involved to limited involvement with research teams, as the new Network was working to build trust and nurture these novel relationships. For most researchers and parent-partners, they have never engaged in a patient-oriented research approach before. There was a steep learning curve for many researchers and patient-partners alike. While effective communication is crucial for both building and maintaining relationships [21], the PPM’s liaison role was essential in identifying emerging needs, clarifying roles and expectations, promoting the use of more accessible language, emphasizing the value of parent-partners’ contributions, and responding to parent-partners’ shifting support needs through the research cycle. For instance, the PPM reviewed surveys that were sent at the Network level to other parent-partners as well as received feedback from them to make more accessible and relevant.

In patient-oriented research, parent-partners’ perspectives are integrated into every stage of the research process from conceptualisation and co-design to data collection, evaluation of results, and dissemination [8]. However, engagement levels and roles of parent-partners can vary from informant to co-lead depending on the goals, knowledge, time commitment, experiences, resources of both parents and researchers as well as project content and phases of the research process [1, 17].

As Amy noticed that some parent-partners were confused with expected roles and their lower engagement of parent-partners in early 2021 in particular, she played the role of informally monitoring the engagement process by regularly reaching out and receiving feedback from parent-partners. Her skills of listening and ability to distill parent-partners’ stories and experiences into a problem that could be solved were applied in this process.

In order to identify the roles that can add value to the research and clarify the process for the incorporation of patient-partners’ input [14, 21, 42], regular evaluation of the engagement process during the life cycle of a research project is a suggested best practice activity [20]. The PPM’s personalized approach was complemented by objective standardized quantitative measures of patient engagement as well as qualitative interviews to enrich our understanding of barriers and facilitators to engagement and strategies that worked well to optimize engagement [10, 11, 13, 19, 32].

Recent patient-oriented research that integrated a patient engagement approach reported the importance of an “engagement coordinator” to prevent and mitigate challenges in building trust and maintaining good working relationships between researchers and patient-partners. The coordinator of research projects could play a role in optimizing engagement by sharing study information with partners (e.g., via email, newsletters, or a project website); offering additional pre- and post-meetings notes or verbal summaries to discuss study progress; integrating partner guidance into study design and conduct; and providing support for patient engagement activities through periodic check-in meetings [21, 22, 30, 33, 34]. However, due to the primary duty of overseeing the conduct of the research project, the coordinator may not be able to dedicate sufficient time for partner engagement. On the other hand, our experiences indicate that the PPM who has lived experience has the ability to directly relate to lived experiences of parent-partners, allowing for trust to happen more quickly.

Furthermore, in order to make a shift in inherent power imbalances between researchers and parent/patient partners in patient-oriented research [7, 25], patient engagement needs to be considered as “a form of partnered negotiation” to create ongoing opportunities for engagement throughout the research process ([26], p. 10). While respect, equitable power sharing, and trust are often cited as foundational principles [20], power differentials are often barriers to open dialogues between researchers and parent-partners. For a subset of researchers, it can be difficult to view patient/parent partners on research teams as equal partners [7]. To address this change, the PPM can be a valuable contributor to attenuate these imbalances by creating a space for parent-partners to bring their voice to a peer, as the PPM was able to identify gaps in expectations from both researchers and parent-partners.

Interpersonal factors such as empathy and supportive relationships, particularly when parent-partners were struggling, can influence the development of trust and an authentic partnership [24]. Our experiences indicate that soft skills, such as empathy, listening, ability to extrapolate the shared concerns and identify the actual problem within the story, are essential to fulfill the PPM role. This role only works in an organisation that is willing to hear, accept and make changes based on the feedback received from interactions with parent-partners. Part of the success of this role stems from having an embedded citizen engagement program, made of patients and caregivers, to support the implementation of issues identified in the PPM interactions and having a network willing to change based on new ideas and ways of working.

Despite the successful individual peer support provided to parent-partners, it was challenging for the PPM to assemble a large group of parent-partners across four time zones to build a virtual community of support. This appeared to be more successful at a project or institution level, where patient and family advisory groups within projects was an effective community-building model [20, 41]. This is an area that deserves further attention in the future.

## Limitations

The current study has some limitations. Despite our purpose of describing diverse perspectives and experiences of parent-partners, we interviewed with only one PPM (Amy), and the accounts documented in Communication Reports are only from parent-partners who wanted to speak to the PPM and also provided their consent to be recorded. Thus, the shared perspectives may not represent those of all parent-partners with diverse experiences within our Network. However, the case study that focuses on engagement with data and in-depth exploration of the phenomena does not seek generalizability based on a sample size [35].

In addition, parent-partners’ accounts were not longitudinal for most individuals, with rare exception, and no direct outcomes in relation to parent-partners’ engagement were assessed by seeking input from parent-partners and researchers regarding the usefulness of their experience with PPMs. We used other approaches to evaluate patient engagement facilitators, barriers and successful strategies and these are described elsewhere [10, 11, 13, 19, 32].

## Conclusion

The current case study highlights the important complementary role that a PPM can play in enhancing patient engagement in research at both individual and network levels. The PPM was able to identify specific challenges experienced by a subset of individuals (e.g., scheduling, accessible language, need for frequent updates on project status, feeling valued) in their efforts to authentically engage as patient-partners in research teams. The PPM was able to suggest effective strategies to enhance engagement, while respecting confidentiality. This allowed the Network to respond by providing more tailored mentorship to research teams through consultation and training initiatives. In addition, concerns raised by parents regarding their interactions with the Network as a whole allowed us to pivot to clarify expectations and compensate parent-partners appropriately for any additional Network-wide activities that they chose to participate in, beyond the research project they had committed to. Future studies evaluating the impact of a PPM in a large research network, including obtaining a first account of parent-partners, and more in-depth analyses of potential power imbalances between groups of parents are warranted. Nonetheless, research institutions and research networks should be encouraged to allocate resources to create and support a PPM/liaison who can assist in supporting parent-partners in their authentic involvement in research teams and provide constructive feedback to the organization on their patient engagement efforts.

### Supplementary Information

**Additional file 1. **GRIPP2-SF.

**Additional file 2. **Interview questions.

**Additional file 3. **CHILD-BRIGHT Guidelines for Patient-Partner Compensation and Recognition.

## Data Availability

The data that support the findings of this study are not publicly available due to privacy and/or ethical restrictions. However, they are available from the CHILD-BRIGHT Network data access committee (admin@childbright.ca) for researchers who meet the criteria for access to confidential data.
