# Supplementary material for: Supporting families and caregivers of children with disabilities through a parent peer mentor (PPM): experiences from a patient-oriented research network
Source: Res Involv Engagem. 2023 Sep 8;9:78. doi: 10.1186/s40900-023-00481-y (PMC10485983; doi:10.1186/s40900-023-00481-y)
Supplement: Supplementary file 1 — Additional file 1. GRIPP2-SF. [file 40900_2023_481_MOESM1_ESM.docx]

GRIPP2 Short Form

| Section and topic | Item |
| --- | --- |
| 1.Aim | Report the aim of PPI in the study   - To describe how a parent peer mentor (PPM) functioned to support involvement of parent-partners in research |
| 2. Methods | Provide a clear description of the methods used for PPI in the study   - The team member included both academic and non-academic (i.e., parent-partners) - One parent peer mentor collected information by contacting parent partners in the Network and documented interactions with parent-partners who provided consent - A research trainee conducted an interview with a parent-peer mentor |
| 3. Study results | Outcomes-Report the result of PPI in the study, including both positive and negative outcomes   - Parent partners provided their input, which helped better understanding of the context and interpretation of emerging themes when the preliminary findings were shared - The PPM created the Figure of the manuscript - Parent partners contributed to edits of the paper during the write-up phase |
| 4. Discussion and Conclusions | Outcomes- Comment on the extent to which PPI influenced the study overall. Describe positive and negative effects.   - Involvement of parent-partners in this study was helpful for researchers to better understand the context of the research Network where the role of the PPM evolved - There were no substantial challenges or negative effects of engagement of parent-partners in this study |
| 5. Reflections/critical perspective | Comment critically on the study, reflecting on the things that went well and those that did not, so others can learn from this experience   - The engagement of two parent-partners in the team was a crucial component of this work, helping researchers better describe both “phenomena” of the PPM and the particular “context” in this case study. The input from the parent-partners contributed to the more nuanced understanding of what “peer” meant to support other parent-partners, most of whom were new to research fields, in the patient-oriented research network. |
