# Supplementary material for: Supporting families and caregivers of children with disabilities through a parent peer mentor (PPM): experiences from a patient-oriented research network
Source: Res Involv Engagem. 2023 Sep 8;9:78. doi: 10.1186/s40900-023-00481-y (PMC10485983; doi:10.1186/s40900-023-00481-y)
Supplement: Supplementary file 2 — Additional file 2. Interview questions. [file 40900_2023_481_MOESM2_ESM.docx]

Additional file 2

**Interview questions for the parent-peer mentor**

Q1. What motivated you to be a parent-peer mentor?

Q2. Looking at the “Parent Peer Mentor at a Glance,” would you pick one change that can highlight the PPM’s contribution and elaborate your experience?

Q3. How did you respond to parent-partners’ comments on the engagement survey?

Q4. Can you tell me about the “Engagement toolbox” in the “Parent Peer Mentor at a Glance”?

Q5. In the communication report, low engagement and some parent-partners’ frustration were mentioned. Can you tell me what was happening during the pandemic? How did you respond to what parent-partners were sharing?

Q6. Why do you think it becomes harder to build a community at a Network level?

Q7. What are the challenges you faced as a parent-peer mentor?

Q8. In some cases, feedback from parent-partners did not lead to make a change. What was behind not seeing change?

Q9. Was there anything that you brought back to parent-partners after communicating with researchers?

Q10. From your perspective, do you have any insights onto how to reach hard-to-reach populations such as low-income/Indigenous families of children with disabilities?

Q11. What are the take-away messages based on your experiences of being a parent-peer mentor?
