# Supplementary material for: Supporting families and caregivers of children with disabilities through a parent peer mentor (PPM): experiences from a patient-oriented research network
Source: Res Involv Engagem. 2023 Sep 8;9:78. doi: 10.1186/s40900-023-00481-y (PMC10485983; doi:10.1186/s40900-023-00481-y)
Supplement: Supplementary file 3 — Additional file 3. CHILD-BRIGHT Guidelines for Patient-Partner Compensation and Recognition. [file 40900_2023_481_MOESM3_ESM.pdf]

CHILD-BRIGHT's Guidelines for Patient-Partner Compensation and Recognition

The purpose of these guidelines is to support the contribution of patient-partners in research, governance and other activities as a member of CHILD-BRIGHT. 'Patient-partners' includes people with lived experience involved in research projects, program committees and other network activities as patients (children, youth, and adults living with a brain-based developmental disability) and/or parents, caregivers, or family members of children, youth and adults living with a brain-based developmental disability.

Patient-partners involved in CHILD-BRIGHT's network activities will be offered compensation in recognition of their contributions.

For each fiscal year, compensation is paid twice yearly, in November and in May. Patient-partners may choose not to be compensated.

Project coordinators and committee chairs must submit annual compensation expectations, including a list of patient-partners, by May 1 of each fiscal year. For example, for fiscal year 2022 (the fiscal year that runs April 1 of the previous calendar year to March 31 of the closing year) requests should be submitted by May 1, 2022.

*Should any patient-partners or activities (for which patient-partners will be compensated) be added to a project or committee plan after May 1, please advise CHILD-BRIGHT immediately.

All requests, questions or concerns can be sent to CHILD-BRIGHT's Citizen Engagement Program Coordinator at citizen.engagement@child-bright.ca.

In order for patient-partners to make an informed choice to participate, research leaders and committee chairs should provide the best estimate of time and effort required specific to the project or committee. Expectations of attendance and contribution, as well as financial considerations and designated compensation should also be communicated in advance of the start of the patient-partner's activity with the project or committee.

Suggested compensation amounts are described in the table below. The amounts reflect the estimated level of engagement required for these different activities.

We are pleased to share our CHILD-BRIGHT Guidelines for Patient-Partner Compensation and Recognition. If you plan to use or adapt part or all of the CHILD-BRIGHT Guidelines for Patient-Partner Compensation and Recognition, we ask that you acknowledge the CHILD-BRIGHT Network.

Role	Commitment	Responsibility	Scope	Suggested Compensation
Member of the research team as a person with lived experience as a patient or family member	Availability by email<br>Willing and able to participate in 3 to 5 meetings by phone or in person per year<br>Attending training may be required	Contributes advice and feedback for decision making by research team	Partnering on a specific research project	$600.00 per year (15-20 hours of work per year)
Additional duties on a research project/committee	Running Zoom rooms<br>Moderating group chats<br>Interviewing participants<br>Other work	Varies	Depends on the work and project	$30 per hour, up to an additional maximum of $600 per year*
Network activities	Completing 2 yearly surveys<br>Reading newsletters & sharing network information Talking with the parent liaison or knowledge mobilization or Communications team (for blog posts, etc.)<br>Attending optional training	Depends on chosen task	Complete 2 surveys and do at least one of the other activities each year	$100 per year (3-4 hours of work per year)

We are pleased to share our CHILD-BRIGHT Guidelines for Patient-Partner Compensation and Recognition. If you plan to use or adapt part or all of the CHILD-BRIGHT Guidelines for Patient-Partner Compensation and Recognition, we ask that you acknowledge the CHILD-BRIGHT Network.

Role	Commitment	Responsibility	Scope	Suggested Compensation
Member of CHILD-BRIGHT committee:<br>-Training & Capacity Building<br>-Knowledge Mobilization<br>-Citizen Engagement Council<br>-National Youth Advisory Panel (NYAP)<br>- Implementation Science Research<br>- Equity, Diversity, Inclusion – Decolonization and Indigenization (EDI-DI)	Commitment to a committee (includes meetings, follow-up actions, etc.)	Participates in decision making by providing options and recommendations	Activity has a network wide mandate	$1,000.00 per year (30-35 hours of work per year)
Member of a working group	Commitment to a group stemming from a program (e.g., Knowledge Mobilization Hubs, Annual General Meeting planning committee, Citizen Engagement Working Group on Engagement Measurement, etc.)	Collaborates with group members towards program/network-related goals or outputs (varies)	Activity has a network-wide mandate	$30 per hour up to a maximum of $600 per year*
Member of a committee as a chairperson or co-chairperson	Chairperson or co-chairperson	Preparing agendas, planning meeting content, leading meetings	Activity has a network-wide mandate	$100 for preparation in addition to the committee honorarium (3-4 hours of work per year)

We are pleased to share our CHILD-BRIGHT Guidelines for Patient-Partner Compensation and Recognition. If you plan to use or adapt part or all of the CHILD-BRIGHT Guidelines for Patient-Partner Compensation and Recognition, we ask that you acknowledge the CHILD-BRIGHT Network.

Role	Commitment	Responsibility	Scope	Suggested Compensation
Member of the CHILD-BRIGHT Network Steering Committee	Contributing member in a governing committee (includes meetings, follow-up actions, etc.)	Has joint responsibility for decision making and mobilizing<br>Initiates and leads activities	Activity has a governing mandate for the network	$1,000.00 per year (30-35 hours per year)
Member of the CHILD-BRIGHT Executive Committee	Contributing member of the executive committee (includes meetings, follow-up actions, etc.)	Has joint responsibility for all network-level activities and budgets	Activity has a governing mandate for the network	1,500.00 per year (48-53 hours per year)
Presenter	Preparing presentation and materials (e.g., slides) and delivering a presentation	Preparation and delivery of a formal presentation, either in person or via a webinar, after working with organizers to ensure alignment with objectives	Making a formal presentation at the CHILD-BRIGHT Annual Meeting or a training/KM session	$100 - $200**<br>Depends on length of presentation and preparation required
Panelist or small group facilitator	Preparing for and performing the particular role as a patient-partner representative	Participation in an informal panel or facilitation of a small group	Being part of an informal panel or facilitating a small group at the CHILD-BRIGHT Annual Meeting or training session	$30 per hour**<br>Pay for preparation, meetings prior, and event itself.
Attendee – conference as CHILD-BRIGHT representative	Representing CHILD-BRIGHT at a SPOR Summit or other event	Attending an event and reporting on it to the appropriate network's Executive member	Active participation at an external event as a patient-partner	$100 per ½ day**<br>$200 per full day

We are pleased to share our CHILD-BRIGHT Guidelines for Patient-Partner Compensation and Recognition. If you plan to use or adapt part or all of the CHILD-BRIGHT Guidelines for Patient-Partner Compensation and Recognition, we ask that you acknowledge the CHILD-BRIGHT Network.

Role	Commitment	Responsibility	Scope	Suggested Compensation
Attendee – mandatory training	Time of training session and any preparation	Check if the training falls outside the scope and hours paid by projects or committees	Attending and participating in the training	$30 per hour ** Maximum of $200 per full day
Engaging children under 10 years of age	For example, reviewing an assent form or interview questions for young children	Should be kept short and reasonable for the age of the individual	Project level	$20 per task (minimum $15 per hour) in gift cards***

We are pleased to share our CHILD-BRIGHT Guidelines for Patient-Partner Compensation and Recognition. If you plan to use or adapt part or all of the CHILD-BRIGHT Guidelines for Patient-Partner Compensation and Recognition, we ask that you acknowledge the CHILD-BRIGHT Network.

*In cases where a patient-partner in a project contributes substantially more over the course of a year than is described under “Commitment” and “Responsibility” in the first row, compensation should be calculated at approximately $30 per hour for the additional work up to a maximum of $600 additionally per year. If the project and the patient-partner agree that the patient-partner’s role and contribution should be compensated at an amount greater than $1,200 per year, the amount over $1,200 should be paid from the project’s budget.

**Patient-partner and/or the researcher must get approval for these activities from the CHILD-BRIGHT Executive Committee prior to the event, or the payment may not be made.

***When engaging children under 10 years of age, consider how the parents will be supporting their child to engage. You may need to consider payment to parents for travel and/or for their role as a support person during meetings depending on the circumstances.

CHILD-BRIGHT suggests a rate of $30 per hour as a guideline for compensation for roles outside of those listed here. Please connect and discuss compensation with patient-partners before the work begins to ensure the expectations are clear on both sides. Any amounts paid by the CHILD-BRIGHT Network not in budget forecasts should be cleared with the Citizen Engagement Program Director before offers are made.

<u>Travel, childcare, and support person expenses</u>

Travel expenses (e.g., airfare, ground transportation, hotel, food) that are reasonable and necessary for participation in CHILD-BRIGHT Network-wide activities such as the annual meeting or face-to-face committee meetings) will be covered in line with the reimbursement policies of the host institution for the project or program administering the patient-partner’s activities. Included in these expenses are those incurred by parents to provide care for their children while they engage in project and network activities.

CHILD-BRIGHT will reimburse patient-partners $15-20 per hour for childcare or support person costs, up to a maximum of $500 per event. If needed, please consult with the Citizen Engagement Program if your childcare or support person costs exceed this amount. Patient-partners must submit a request for reimbursement for travel and childcare/support person costs in advance of the event and for receipts (or other appropriate documentation) after the event.

Compensation for these activities, if pre-approved, will be covered by the CHILD-BRIGHT Network (central office budget).

We are pleased to share our CHILD-BRIGHT Guidelines for Patient-Partner Compensation and Recognition. If you plan to use or adapt part or all of the CHILD-BRIGHT Guidelines for Patient-Partner Compensation and Recognition, we ask that you acknowledge the CHILD-BRIGHT Network.

Note: Patient-partners should be advised that compensation (including money and/or gift cards) received as compensation or recognition of their participation in network activities are subject to relevant tax laws and must be reported on both federal and provincial tax forms. Research leaders or committee chairs should inform patient-partners that compensation has the potential to impact a patient's financial situation and inform them that it is their responsibility (the patient-partner's responsibility) to determine whether payment is the right fit for them.

Note: Patient-partners cannot request payment from CHILD-BRIGHT if they work for an organization that also pays them for those same hours worked.

These guidelines are reviewed and updated by the CHILD-BRIGHT Citizen Engagement Council, the NYAP and the Network Steering Committee yearly, at a minimum.

Considerations in patient-partner recognition

In addition to compensation, research leaders and committee chairs should also carefully consider what recognition would be appropriate for their patient-partners (e.g., named on grant applications, websites, presentations, and research articles).

It is also important for research leaders and committee chairs to clearly inform patient-partners that anonymity may not be possible when being part of a research team, unlike when they are a participant in a research project. Being a member of a research team means patient-partner names will be included in grant applications and on project materials, as many grants, competitions, and even scientific journals require patient engagement.

Note: Patient-partners should consider this carefully as public recognition may open up ethical implications for their family, since it often reveals personal health information about their child or family. Research leaders and committee chairs should keep track of where patient-partners agree to have their names released and should collect consent prior to publishing or disclosing any details shared by the patient-partner about their family or personal journey.

When discussing certain recognition activities, research leaders and committee chairs should ensure that patient-partners understand what additional work may be required and understand if that work will be compensated. For example, being named as an author in a research article requires following strict journal authorship guidelines, including giving feedback on drafts and reviewing and approving the final version.

We are pleased to share our CHILD-BRIGHT Guidelines for Patient-Partner Compensation and Recognition. If you plan to use or adapt part or all of the CHILD-BRIGHT Guidelines for Patient-Partner Compensation and Recognition, we ask that you acknowledge the CHILD-BRIGHT Network.

As with most work with patient-partners, preferences may vary widely between partners, so research leaders and committee chairs must ensure to collect consent and keep channels of communication open.

We are pleased to share our CHILD-BRIGHT Guidelines for Patient-Partner Compensation and Recognition. If you plan to use or adapt part or all of the CHILD-BRIGHT Guidelines for Patient-Partner Compensation and Recognition, we ask that you acknowledge the CHILD-BRIGHT Network.
